# Effects of Wireless Remote Microphone on Speech Recognition in Noise for Hearing Aid Users in China

**DOI:** 10.3389/fnins.2021.643205

**Published:** 2021-04-12

**Authors:** Jing Chen, Zhe Wang, Ruijuan Dong, Xinxing Fu, Yuan Wang, Shuo Wang

**Affiliations:** Beijing Institute of Otolaryngology, Otolaryngology—Head and Neck Surgery, Beijing Tongren Hospital, Capital Medical University, Beijing, China

**Keywords:** hearing aids, sensorineural hearing loss, signal-to-noise ratio, wireless remote microphone, speech-in-noise recognition

## Abstract

**Objective:** This study was aimed at evaluating improvements in speech-in-noise recognition ability as measured by signal-to-noise ratio (SNR) with the use of wireless remote microphone technology. These microphones transmit digital signals via radio frequency directly to hearing aids and may be a valuable assistive listening device for the hearing-impaired population of Mandarin speakers in China.

**Methods:** Twenty-three adults (aged 19–80 years old) and fourteen children (aged 8–17 years old) with bilateral sensorineural hearing loss were recruited. The Mandarin Hearing in Noise Test was used to test speech recognition ability in adult subjects, and the Mandarin Hearing in Noise Test for Children was used for children. The subjects’ perceived SNR was measured using sentence recognition ability at three different listening distances of 1.5, 3, and 6 m. At each distance, SNR was obtained under three device settings: hearing aid microphone alone, wireless remote microphone alone, and hearing aid microphone and wireless remote microphone simultaneously.

**Results:** At each test distance, for both adult and pediatric groups, speech-in-noise recognition thresholds were significantly lower with the use of the wireless remote microphone in comparison with the hearing aid microphones alone (*P* < 0.05), indicating better SNR performance with the wireless remote microphone. Moreover, when the wireless remote microphone was used, test distance had no effect on speech-in-noise recognition for either adults or children.

**Conclusion:** Wireless remote microphone technology can significantly improve speech recognition performance in challenging listening environments for Mandarin speaking hearing aid users in China.

## Introduction

With the advancement and evolution of current hearing technology, a variety of digital audio electronic devices have become more prevalent in the general population. These accessories which allow wireless transfer of high quality audio, such as smartphone compatible wireless earphones, have enhanced listening experiences for normal-hearing listeners, with the potential of similar accessories improving listening experiences for the hearing-impaired as well (Picou, 2020).

Sensorineural hearing loss not only reduces the sensitivity to sound and dynamic range of auditory perception for hearing-impaired listeners, but also reduces their frequency and temporal resolution that can lead to difficulty in speech comprehension (Moore, 2013). Hearing aids have been proven to be an effective solution in compensating for hearing loss in the loudness domain, but cannot compensate sufficiently for issues with frequency or temporal resolution. These issues become more pronounced in listening environments where the target sounds are masked by competing sounds. An effective way to help people with sensorineural hearing loss in more challenging listening environments is to improve the audibility of the target signal. The signal-to-noise ratio (SNR) is defined as the ratio of speech signals to noise and is frequently used to indicate the quality of the target signal in challenging communication environments. Research has shown that the speech-in-noise recognition ability in people with sensorineural hearing loss is significantly reduced when the SNR is at or below 5 dB. In contrast, the speech-in-noise recognition performance for normal-hearing listeners are minimally impacted at a SNR of 0 dB (Dong et al., 2015). Wilson et al. (2007) reported that individuals with a moderate hearing loss required an increased SNR of up to 10 dB to achieve the same speech understanding as individuals with normal hearing. Generally speaking, SNR depends on three key determinants: the presence of background noise, the distance between the listener and the target signal, and the reverberation in the listening environment. If the competing noise level is stable and the distance between the listener and target speech increases, the effective SNR for the listener will decrease. Studies have shown that to achieve better speech signal clarity, the distance between the listener and the signal source should be no greater than 1.8 m (Fickes, 2003). Blazer (2007) reported that students with hearing loss were able to achieve 95% on speech recognition tasks when they were 1.8 m apart from the source of interest, and only 60% when they were 7.3 m apart from the source of interest. In addition to the target distance, Reverberation Time (RT) plays an important role as well: the longer the RT in the communication environment, the more difficult it is for people with sensorineural hearing loss to communicate. Studies have shown that reducing reverberation time from 1.2 to 0.3 s can lead to 11 dB improvement in SNR (David and William, 1984). Furthermore, some studies have shown that optimal SNR for speech perception is dependent on a child’s age, with younger children requiring a more favorable SNR to obtain similar speech recognition scores as adults. Adult-like performance is reached at the age of 10–12 years in stationary noise conditions (Koopmans et al., 2018).

Hearing aids today can provide listeners with a clear, high-quality sound experience in a quiet environment, but they deteriorate in the presence of noise (Bentler et al., 2016). Kochkin (2002) found that nearly 45% of hearing aid users were dissatisfied with their hearing aid performance in a noisy environment. One of the main goals of the development of current hearing aid technology is to improve speech recognition in complex listening environments and improve SNR in conditions where noise, distance, and reverberation can interfere. One of the technologies, directional microphones, can significantly improve speech recognition in noise for specific listening environments. Directional microphones perform best when the speech is presented from the front, the noise is in the rear, and the target speaker is in close proximity. However, in a real-world communication environment, directional microphones may fall short as these conditions are often not met (Kreikemeier et al., 2013; Picou et al., 2014).

When hearing aids and their microphones alone do not provide adequate assistance, some of the most beneficial wearable technology comes in the form of assistive listening devices which can also effectively improve the SNR for hearing aid users. For example, remote microphone hearing assistance technology (HAT) is widely used for hearing-impaired children. Amongst various HAT devices, wireless frequency modulation (FM) system is an early development that is still widely used. A typical FM unit consists of a small transmitter and microphone, which picks up the voice of a speaker and sends the clean speech to a radio-frequency (RF) receiver plugged into the hearing aid of a listener. Using a FM system to transmit the teacher’s voice directly to the student is equivalent to reducing the communication distance to within 3–6 inches. Boothroyd showed that using the FM system in a noisy environment resulted in the same speech recognition as in a quiet environment (Boothroyd and Guerrero, 2004). For FM systems, American Speech-Language-Hearing Association (ASHA) recommends, “… the basic goal is that the FM system should increase the level of the perceived speech, in the listener’s ear, by approximately 10 dB” [American Speech-Language-Hearing Association (ASHA), 2002]. Lewis et al. (2004) reported that on average, subjects improved by 14.2–16.7 dB with the use of one FM receiver over the use of two hearing aids alone in the directional microphone mode. Current hearing aids often utilize digital radio frequency technology, such as Bluetooth Remote Microphones, which wirelessly connect to hearing aids. These devices function similarly to FM systems and can wirelessly transmit audio signals to hearing aids over long distances (up to 10 m). In most hearing aid applications, this technology does not require an extra receiving device like a traditional FM system, as the digital wireless antenna is built into the hearing aids. This makes them more convenient to carry and operate than traditional FM systems. Research has clearly indicated that the use of remote microphone systems statistically improved speech recognition in noise, relative to unaided and hearing aid-only conditions for adults with hearing loss (Jace et al., 2015; Rodemerk and Galster, 2015).

Mandarin Chinese is a tonal language with four phonologically distinctive tones characterized by syllable-level fundamental frequency (F0) contour patterns. These pitch contours are commonly described as high–level (tone 1), high–rising (tone 2), falling–rising (tone 3), and high–falling (tone 4) (Lin, 1988). According to a study of the hearing disabled population from four provinces in 2016, about 5% of the population in mainland China have hearing impairment (Hu et al., 2016). However, it was speculated that only about 7–10% of those hearing impaired listeners have been fitted with hearing aids (Zhang, 2009), suggesting a large unreached population of hearing-impaired Chinese listeners that could benefit from the use of hearing aids and the assistive listening devices. Previous studies (Jace et al., 2015; Rodemerk and Galster, 2015; Bentler et al., 2016) have shown that the remote microphone HAT can significantly improve the speech recognition ability of the hearing-impaired people who communicate in English in noise. However, there are few reports on the application of this technology in the hearing impaired population who speak Mandarin Chinese. It is our interest to investigate how much improvement Chinese hearing-impaired listeners may benefit from the current wireless remote microphone technology. This study was aimed at evaluating improvements in speech-in-noise recognition ability as measured by signal-to-noise ratio (SNR) with the use of wireless remote microphone technology.

## Materials and Methods

### Subjects

**FIGURE 1:**
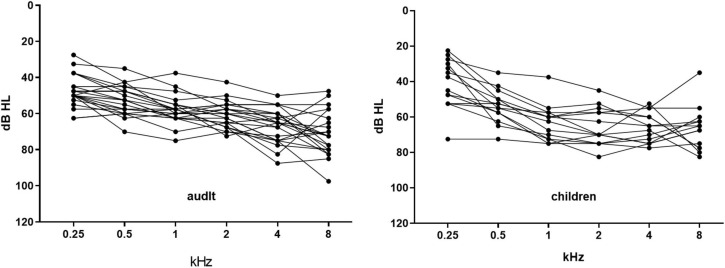
The average hearing thresholds between the two ears of each individual adult and child. The horizontal axis represents the frequency. The vertical axis represents the hearing thresholds in dB HL. Each dot represents the average threshold of the left and right ears at this frequency of one individual.

Two groups of subjects were recruited in this study. All subjects had a history of digital hearing aids use for more than 1 year but no experience with HAT in combination with their hearing devices. Twenty-three native Mandarin Chinese-speaking adult subjects (6 females and 17 males) participated in the adult group. The subjects were between 19 and 80 years old (Mean = 63.4, *SD* = 18.7) and had relatively symmetric sensorineural hearing loss in both ears. The mean pure-tone hearing thresholds at frequencies of 500, 1,000, 2,000, and 4,000 Hz (PTA_0.5 to 4 kHz_) across the two ears for the groups of subjects ranged from 40 to 75 dB HL, as shown in Figure 1.

Fourteen native Mandarin Chinese-speaking children (7 females and 7 males) were recruited for the children’s group. These subjects were between 8 and 17 years old (Mean = 13.1, *SD* = 3.2), and had bilateral sensorineural hearing loss with a PTA_0.5 to 4 kHz_ ranging from approximately 35 to 80 dB HL, as shown in Figure 1.

### Hearing Aid Fitting Equipment

In this study, adult participants were fitted with two ReSound LiNX2 962 Receiver-In-The-Ear (RIE) hearing aids, and pediatric participants were fitted with two ReSound UP 988 Behind-the-Ear (BTE) hearing aids. Pediatric participants utilized their own earmolds during the test, which were coupled to hearing aids. Pediatric subjects with good low frequency hearing had earmolds with small vents., which would have negligible effect on the gain of the amplified sound path (Dillon, 2012). A ReSound Mini Microphone was paired to the test hearing aids in all cases. The ReSound Mini Microphone is a small personal streaming device which utilizes 2.4 GHz digital wireless technology to transmit sounds from the remote microphone or the output signal from any external audio source, directly to a Resound hearing aid. The remote microphone can be clipped onto the speaker’s clothing or placed on a surface to transmit the voices of multiple speakers. It provides a wireless link between the speaker and the listener with no additional hardware or connections required. The audio frequency range of the Mini Microphone is from 100 to 8,000 Hz. A remote control was used to change the hearing aid program during the test. The ReSound Aventa 3.10 software was used to fit hearing aids for subjects.

### Test Equipment and Materials

The test equipment included five Audioengine2 + active speakers, four of which were used to present noise signals and one was used to present the target speech. The Mandarin Hearing in Noise Test (MHINT) was used to test the speech recognition ability for adults. The HINT is a standardized and recorded test that can be used to estimate the signal-to-noise ratio (SNR) at which the sentences embedded in background noise can be repeated correctly 50% of the time. MHINT is the Mandarin version of HINT. The MHINT materials consist of 12 lists, each containing 20 sentences. Each sentence contains 10 Chinese characters. The presentation level is response dependent; lowered or raised according to a participant’s correct or incorrect response of the test material. Presentation levels were decreased by 2 dB after a correct response and increased by 2 dB after an incorrect response. The reception threshold for sentences (RTSs) was calculated using this adaptive procedure (Wong et al., 2007). The Mandarin Hearing in Noise Test for Children (MHINT-C) was used to test the children. The MHINT-C materials consist of 12 lists, each containing 10 sentences (Chen and Wong, 2020). For each test condition, a list of 10 MHINT sentences were presented in a randomized order. Speech-shaped noise (SSN) was used as the masker noise in the present study.

### Test Environment

**FIGURE 2:**
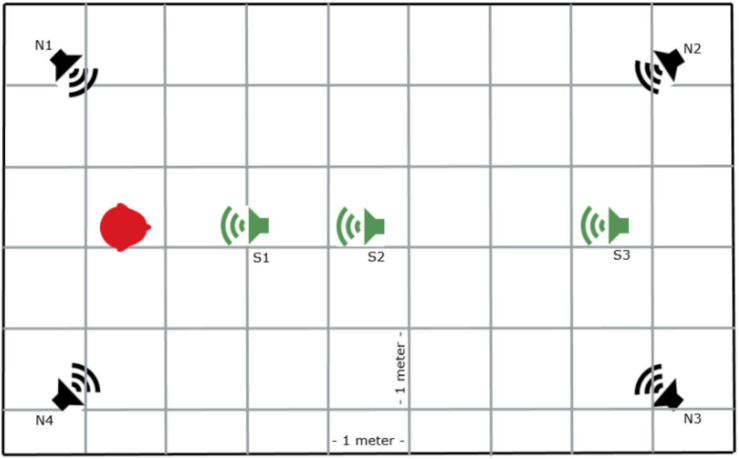
Five speaker set up in the testing room. Four speakers playing the noise placed in the four corners (N1–N4) of the test room, and 0.75 m from the wall corner. The speaker playing speech signals were located at S1, S2, and S3, respectively. S0 is the location of the subject (Red mark).

The test location was a quiet meeting room measured at 5.5 × 8 × 2.5 m, with ambient background noise levels below 45 dB A. The testing room resembles a typical classroom setting with a reverberation time of 0.46 s. As shown in Figure 2, four speakers were utilized for the noise located at the four corners of the room, 0.75 m away from the walls in the corners (N1–N4) of the test room, 45 degrees away from the two vertical walls, facing the center of the room. The participants were seated at the S0, 1.5 m away from the back wall. The speakers presenting speech signals were located at 0°azimuth distanced at 1.5, 3, and 6 m directly in front of the participant (S1, S2, and S3 conditions, respectively), resulting in a critical distance of 1.41 m. The wireless remote microphone was set to directional and clipped to the participant’s collar. In the test, the wireless remote microphone was suspended 25 cm below the speaker to simulate the distance and orientation between the speaker’s mouth and the Mini Microphone in practical applications. The speaker was set at ear level for each participant. Four speakers labeled N1, N2, N3, and N4 were used to deliver speech-shaped noise simultaneously at a calibrated noise level of 65 dB A at S0.

### Test Procedures

Adult subjects were fitted with ReSound LiNX2 962 RIE hearing aids on both ears according to their audiograms. The proprietary fitting prescription of Audiogram + in the ReSound Aventa 3 software was used. The hearing aid microphones were set to a fixed directional response, and all other advanced signal processing features (e.g., directional processing, digital noise reduction, wind noise reduction, reverberation processing, frequency lowering) were disabled. The ReSound Mini Microphone was paired with the hearing aids and set at a hearing aid to Mini Microphone ratio of 1:1. (i.e., remote microphone and hearing aid microphone were set such that each contributed equally to the output audio signal). There were nine test conditions consisting of three test distances and three program settings. Each program setting was tested at each distance condition. The program settings included directional microphones active (HA_D), the Mini Microphone active only (MM), and both hearing aid microphones and MM active (HA_D + MM). The nine test conditions were carried out in a randomized order. The remote control was used by the investigator to switch the hearing aid program settings. For each test condition, a list of MHINT materials were presented to obtain the speech recognition threshold in SNR. During the test, the SSN noise level was fixed at 65 dB A.

Pediatric subjects were fitted with bilateral Resound UPS988-DLW BTE hearing aids based on their audiograms with a DSL v5 fitting prescription. The programming of the hearing aids were the same as those utilized for the adult subjects, with the addition of an omnidirectional microphone mode (HA_O) program. Hence, for the pediatric subjects, there were twelve test conditions (four test program settings at three distances each) carried out in a randomized order. For each test condition, a list of MHINT-C materials were used to obtain recognition thresholds in SNR.

### Statistical Methods

Statistical analysis was carried out using Statistics Package for Social Science (SPSS) 16.0. A repeated-measures analysis of variance (RM-ANOVA) was conducted to determine if there was any overall statistical significance among the outcome SNRs across the three or four device settings at the three test distances for both adult and pediatric groups. The test distances and device settings were considered the independent variables. SNR was considered the dependent variable.

## Results

### Speech Recognition for Adults

The SNRs obtained under nine test conditions for adults are detailed in Table 1. The RM-ANOVA revealed a statistically significant difference among each device setting [*F*(2, 65) = 267.91, *p* < 0.05]. There was a significant interaction effect between the distance and device settings [*F*(4, 62) = 7.77, *p* < 0.05]. This indicates that SNR is affected by the interaction between the distance and device settings.

**TABLE 1:** The speech in noise recognition thresholds in SNRs (dB) obtained under each test condition for both adults and children.

**Adult**				**Children**			
	**HA__D**	**HA + MM**	**MM**	**HA__0**	**HA__D**	**HA + MM**	**MM**
1.5 m	5.55 ± 5.90	−2.23 ± 6.87	−5.46 ± 7.50	4.09 ± 5.56	1.66 ± 1 ± 6.19	−4.37 ± 5.66	−9.26 ± 1 ± 5.11
3 m	11.77 ± 6.90	−3.30 ± 6.99	−5.88 ± 1 ± 6.71	8.16 ± 1 ± 5.28	6.77 ± 6.12	−3.66 ± 7.23	−9.65 ± 4.55
6m	13.11 ± 7.08	−4.87 ± 5.96	−7.53 ± 7.08	10.16 ± 8.92	8.47 ± 8.14	-4.47 ± 8.10	−10.38 ± 5.00

**FIGURE 3:**
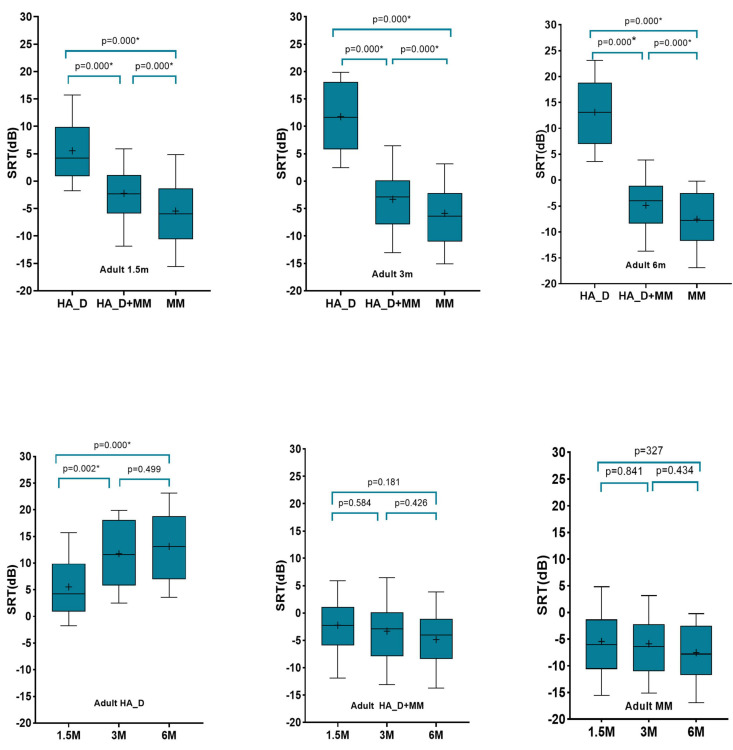
Comparison of speech in noise recognition threshold (measured in SNR) of adult subjects with different test distances and different device settings. Boundaries of the boxes indicate the 25th and 75th percentile. Whiskers indicate the 10th and 90th percentiles. Solid lines denote the median. Plus denotes the mean.

Figure 3 illustrates the speech-in-noise recognition threshold (measured in SNR) of adult subjects with different test distances and different test device settings. The results showed that at the same test distance, the SNR thresholds under three device settings were significantly different from each other (*p* < 0.05). Performance was significantly better when the MM was active compared to the hearing aid microphone alone (*p* < 0.05). The performance with the MM alone was significantly better than the performance with HA_D + MM (all *p* < 0.05).

Moreover, the results showed that with the hearing aid microphone alone, the SNR for the 1.5 m condition was significantly better than those for the 3–6 m conditions (*p* < 0.05), with no significant differences in SNR between 3 and 6 m conditions. When the MM was active, the test distance had no effect on SNRs (*p* > 0.05).

### Speech Recognition for Children

The SNRs obtained under twelve test conditions for children are detailed in Table 1. The RM-ANOVA revealed a statistically significant difference among each device setting [*F* (2, 65) = 267.91, *p* < 0.05]. There was a significant interaction effect between test distance and device settings (*p* < 0.05). This indicates that the SNR is affected by the interaction between the distance and device settings.

**FIGURE 4:**
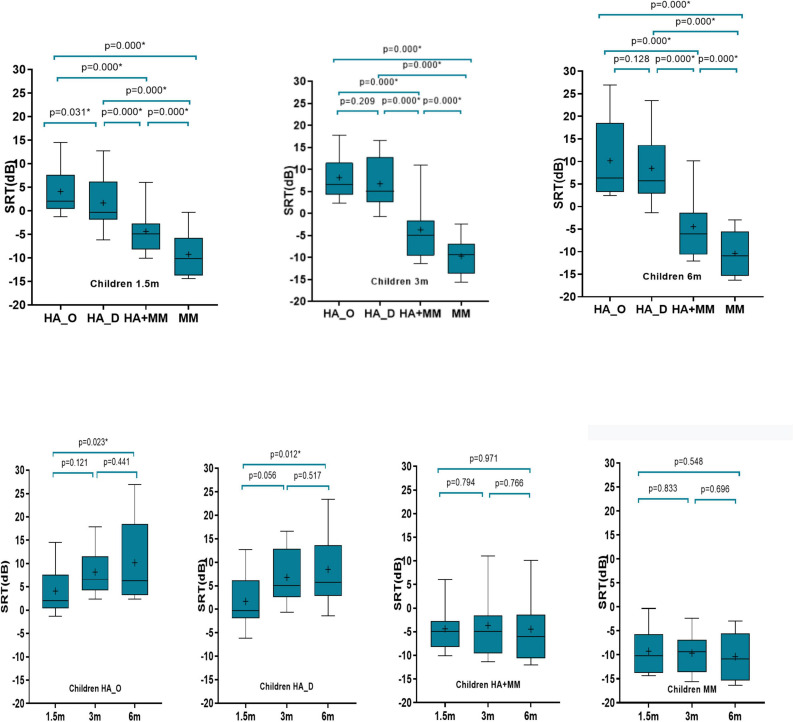
Comparison of speech in noise recognition threshold (measured in SNR) of pediatric subjects at different test distances and different test device settings. Boundaries of the boxes indicate the 25th and 75th percentile. Whiskers indicate the 10th and 90th percentiles. Solid lines denote the median. Plus (+) denotes the mean.

Figure 4 illustrates the speech-in-noise recognition threshold (measured in SNR) of children subjects with varying test distances and test device settings. The results showed that the SNR thresholds under 1.5 m conditions for four device settings were significantly different from each other (*p* < 0.05). Performance was significantly better for the two conditions with the MM active, in comparison with the hearing aid microphone alone (*p* < 0.05) regardless of microphone directionality. The performance for the MM alone was significantly better than the performance with MM + HA (all *p* < 0.05). When the test distance was set at 3 and 6 m, there were no differences in performance between HA_O and HA_D.

The results show that using the hearing aid microphone alone, regardless if directional or omnidirectional, the SNR in the 1.5 m condition was significantly better than that for the 6 m condition, with no significant difference in SNR between 1.5 and 3 m, 3 and 6 m. When the MM was active, the test distance had no effect on speech in noise recognition thresholds (*p* > 0.05).

## Discussion

A remote microphone can be connected with a hearing aid and/or a cochlear implant to improve the speech recognition ability for patients with sensorineural hearing loss in noise. In a study of children with moderate to severe sensorineural hearing loss wearing bilateral hearing aids, Lewis et al. investigated the effects of remote microphone technology on speech perception in noise relative to hearing aid only conditions. Results revealed that the use of bilateral FM audio streaming significantly improved SNR relative to the omnidirectional hearing aids alone by 16–22.7 dB, confirming that adult listeners with hearing loss benefit from the use of a personal remote microphone system (Lewis et al., 2004). Research by Jantien et al. (2017) showed that using a wireless remote microphone in a noisy environment improved Speech Reception Thresholds (SRTs)in adults with bilateral cochlear implants by 5.4 dB. In a study with preschool children using remote microphones as personal wireless systems with cochlear implants, Clare and Gill (2018) showed that with professional guidance and training at home, this technology has the potential to improve education and communication environments for preschool children. In the present study, the benefit of using the remote microphone was consistent in all three test distances for both adults and children. Speech in noise recognition thresholds, measured in SNR, at all test distances decreased significantly, indicating a significant improvement in the speech recognition performance in noise. Compared with the HA_D setting, the results with the MM improved by 11–19.5 dB for adults and by 10–18.9 dB for children. The benefits of adding the MM compared to the HA alone increased as the test distance increased. Regardless of whether the HA was set to omnidirectional or fixed directional, increases in distance resulted in a rapid decrease in SNR for the hearing aid microphones only condition. In the conditions where MM was used, the distance between the MM and sound source remained constant even though the listener wearing hearing aids moved further away. Thus, the SNR at the location where the target speech was detected remained the same. The use of a wireless remote microphone could very well improve the problem of reduced signal-to-noise ratios due to greater distances by increasing the desired sound in communication environments.

In this study, we found that for both adult and pediatric subjects, speech in noise recognition thresholds using the MM alone were significantly better than using HA_D + MM. This finding is similar to Linda et al., who reported that when using a FM-only microphone setting, the SNR is determined primarily by the SNR of the FM microphone; when both HA and FM microphones are active, the SNR is determined by the highest level of the speech, which is typically at the FM microphone, and the highest level of noise at either the FM or HA microphone. Linda showed that better performance was observed in the FM-only compared to FM + HA condition. The amount of improvement in the SNR is determined by the levels of noise at the FM and HA microphones. When the noise levels are similar at the two microphones, an improvement in the SNR of 2 dB is expected (Linda and Kristen, 2016). In the present study, the use of MM alone could provide 3 dB improvement in SNR compared to HA_D + MM settings for adult subjects, and 6.5 dB improvement for pediatric subjects. This phenomenon was more distinct in children. Compared with adults, it is difficult for children to concentrate on listening tasks in low SNR conditions (Ryan and Patricia, 2011). Therefore, the negative impact of low SNR listening environments on children is greater than that seen in adults. The results of this study also showed that children are more likely to experience “floor effects” than adults in hearing aid microphone only conditions.

It has been established that the use of hearing aids with directional microphones can improve speech communication in noise for people with sensorineural hearing loss; however, varying degrees of improvement have been reported. Early research showed that directional microphones can improve speech in noise by 6–8 dB compared to omnidirectional microphones (Gravel et al., 1999; Kuk et al., 1999). Ricketts et al. performed HINT tests on 47 adults using five different hearing aids to evaluate the advantages of directional microphones. Speech was presented from a 0° azimuth with simulated cafeteria noise presented from 30°, 105°, 180°, 225°, and 330° azimuths (Ricketts et al., 2001). An average directional benefit of 2.2–2.9 dB compared to omnidirectional microphones was reported (Ryan and Patricia, 2011). In the present study, when the test distance was 1.5 m, the directional microphone responses were significantly better than omnidirectional responses for children. When the test distance increased to 3 and 6 m, there were no significant differences among the directional and the omnidirectional microphones. The directional microphone advantage disappeared, the possible reason being that the increase in test distance led to the rapid decline of signal-to-noise ratio, resulting in the “floor” effect. The directional microphone loses its advantage under the condition of low signal-to-noise ratio, which leads to no significant differences among the directional and the omnidirectional microphones.

In this study, when listening via the hearing aid microphones only, speech in noise recognition performance in adult subjects decreased as the test distance increased from 1.5 to 3 m. However, in children subjects, the same trend as that of adults was observed, but did not reach a standard of statistical significance. The consideration may be related to the small number of children subjects. Whether adult or child subject, no further decrease was observed when the test distance increased from 3 to 6 m. This was not a surprising finding as performance often decreases with increasing distance (Wilson et al., 2007). The lack of a further decrease between the 3 and 6 m test distances could be due to a “floor effect” where decreased signal levels at further distance did not result in an even poorer speech in noise recognition performance, perhaps due to reverberation and distortion of the speech signal caused by the test environment.

Compared with English, most initial consonants in Mandarin are voiceless. This results in initial consonants with low sound intensities as voiceless signals do not entail the vibration of the vocal folds and makes Mandarin comparatively more difficult to recognize in noise. This study showed that the Mini Microphone can effectively improve speech communication in Mandarin-speaking patients with sensorineural hearing loss.

Currently, multiple hearing aid manufacturers have introduced digital wireless remote microphones compatible with their range of hearing aid and cochlear implant technologies. Operation of these devices is simple, could be easily adopted by hearing aid users, especially older adult users, and adds relatively little cost to the hearing aid purchase. For future studies, a comparative study of speech intelligibility, speech delay, and cumulative power consumption of multiple digital wireless devices used in conjunction with hearing aids may be considered. Lastly, the Bluetooth SIG (Special Interest Group) has introduced a digital wireless standard for manufacturers of hearing aids and wireless accessories, as well as consumer devices. With the increase of such technology, Bluetooth digital signal coverage in public spaces such as theaters and cinemas may increase, improving accessibility for hearing aid users who may be able to use remote microphones in public spaces to improve communication and listening.

In the current study, loudspeakers were used as the source of speech signals. The role of lip-reading and facial expressions in communication was not fully examined. However, in daily communication, lip reading and facial expressions play a vital role in understanding speech, especially for hearing-impaired individuals and children (Chodosh et al., 2020). The results of this study showed that MM alone provided the best speech recognition ability in a noisy environment for both adults and children, but this result should not be interpreted as a basis to deactivate a hearing aid microphone in noisy environments. For hearing-impaired children, hearing aid microphones can increase the chances of incidental learning (Vermeulen et al., 2012; Klein et al., 2018). HA + MM may be considered as a part of a more comprehensive program, where both target speech and incidental learning are desired.

Lastly, one of the limitations of the current study is that, only native speakers of Mandarin Chinese were selected. For future studies, bilingual (e.g., Mandarin Chinese and English) adults and children could be recruited to evaluate the effect of remote microphone and assistive listening devices in both tonal and non-tonal languages. In addition to the aforementioned wearable assistive listening devices, speech-to-text conversion apps for smart phones have been designed specifically to provide communication redundancy for individuals with hearing loss. These apps have been shown to improve communication for those with hearing loss, especially the profoundly hearing impaired population in certain listening situations (Pragt et al., 2020).

## Conclusion

The addition of a wireless remote microphone to bilaterally worn hearing aids compensates for increased distance from the sound source. The use of a wireless remote microphone can significantly improve speech in noise communication performance in Chinese hearing-impaired listeners.

## Data Availability Statement

The original contributions presented in the study are included in the article/supplementary material, further inquiries can be directed to the corresponding author/s.

## Ethics Statement

The studies involving human participants were reviewed and approved by the Institutional Review Board at the Beijing Institute of Otolaryngology and Beijing Tongren Hospital. Written informed consent to participate in this study was provided by the participants’ legal guardian/next of kin.

## Author Contributions

SW, JC, and RD contributed conception and design of the study. JC, RD, ZW, and YW performed the experiments. JC, SW, XF, and RD performed the statistical analyses. JC and SW wrote the first draft of the manuscript. All authors contributed to manuscript revision, read, and approved the submitted version.

## Conflict of Interest

The authors declare that the research was conducted in the absence of any commercial or financial relationships that could be construed as a potential conflict of interest.
